# Bibliometric and visual analysis of membranous nephropathy literature from 2010 to 2023

**DOI:** 10.3389/fphar.2024.1426897

**Published:** 2024-09-12

**Authors:** Yirui Chen, Chen Liu, Hongnan Shen, Pingping Su, Liang Pang, Congcong Zeng, Jinguo Cheng

**Affiliations:** ^1^ Wenzhou Hospital of Traditional Chinese Medicine, Affiliated with Zhejiang Chinese Medical University, Wenzhou, China; ^2^ The Third School of Clinical Medicine, Zhejiang Chinese Medical University, Hangzhou, China; ^3^ The First Affiliated Hospital of Wenzhou Medical University, Wenzhou, China

**Keywords:** membranous nephropathy, membranous glomerulonephritis, a2 receptor antibody, bibliometric analysis, citespace, VOSviewer

## Abstract

**Background:**

Membranous glomerulonephritis, also known as membranous nephropathy (MN), is a common cause of nephrotic syndrome in adults. Despite extensive research on MN, bibliometric studies on the subject are scarce. Therefore, this study aimed to provide a visual analysis of global trends in membranous nephropathy research over the past 13 years.

**Methods:**

This study conducted a bibliometric and visual analysis of global trends in MN research from 2010 to 2023. Articles related to MN were retrieved from the Web of Science Core Collection (WoSCC) database. Tools such as CiteSpace and VOSviewer were utilized to analyze publications, countries, institutions, authors, publishing journals, co-cited references, and keywords to identify the current state and future trends in MN research.

**Results:**

The analysis encompassed 1,624 publications, showing an annual increase from 2010 to 2023. The People’s Republic of China emerged as the most active country in this field, while France’s Sorbonne Universite and Institut National de la Sante et de la Recherche Medicale (Inserm) led in publication volume among academic institutions. Debiec Hanna stood out as the most prolific author. BMC Nephrology had the highest number of publications, making it the most favored journal in the field. The article with the greatest co-citation intensity was “Primary Membranous Nephropathy,” a review published in 2017.

**Conclusion:**

This study shows that there has been increasing interest in membranous nephropathy over the past 13 years. The most frequently encountered keywords were “membranous nephropathy” “nephrotic syndrome,” and “glomerulonephritis.” Analysis of emerging terms indicated that “a2 receptor antibody,” “domain containing 7a,” and “t cell” may remain prominent subjects of research in the forthcoming years. The findings highlight key research trends and areas of interest that can inform researchers, clinicians, and policymakers about the current state of MN research and help guide future research directions and clinical practice.

## 1 Introduction

Membranous nephropathy (MN) is the most common cause of idiopathic nephrotic syndrome in non-diabetic adults (Couser, 2017). While it can occur at any age, it is predominantly seen in adults, with a peak incidence between 36 and 40 years and a male to female ratio of 2:1 (Couser, 2017). MN often begins insidiously, with some cases preceded by an infection (Ronco et al., 2021). The initial symptom is usually a gradually worsening swelling of both lower limbs, with most cases presenting significant proteinuria and manifesting as nephrotic syndrome (Liu et al., 2016). MN is thought to result from glomerular damage caused by autoantibodies against antigens on the membranes of normal podocytes. It is characterized by the deposition of subepithelial immune complexes and thickening of the basement membrane, visible under light and electron microscopy, often forming spikes (Infantino, 2022). The evidence suggests the immune deposits include Immunoglobulin G and significant antigens such as phospholipase A2 receptor (PLA2R) (Yang et al., 2021) and thrombospondin type-1 domain-containing 7A (THSD7A) (Tomas et al., 2014).

Bibliometrics is a method that applies mathematical and statistical techniques for qualitative and quantitative analysis of publications within a specific field. This approach utilizes data analysis to evaluate the current research landscape, identify hot topics, and forecast the future direction of a discipline, offering a timely and intuitive means for delving into specific areas (Ninkov et al., 2022). As of now, there has been no bibliometric analysis conducted on MN. Hence, this study aims to review existing research and perform a systematic analysis to explore the state and trends of MN from 2010 to 2023. Using bibliometric analysis, with CiteSpace as the primary tool and VOSviewer as a supplement, this study examines publications, countries, institutions, authors, publishing journals, co-cited references and keywords within the field of MN. The goal is to summarize the current state of research and development, providing guidance for future research directions.

## 2 Materials and methods

### 2.1 Data source and search strategy

The Web of Science Core Collection (WoSCC) was utilized for data collection. Additionally, to ensure retrieval accuracy, we used topic retrieval and set the search terms to TS = (“membranous nephropathy” OR “idiopathic membranous nephropathy” OR “primary membranous nephropathy” OR “membranous glomerulonephritis”). The search concluded on 30 January 2024. Included publications were primarily articles and reviews, and the literature language was restricted to English. The data were saved and stored in the Plain Text File format.

### 2.2 Data extraction and processing

**FIGURE 1:**
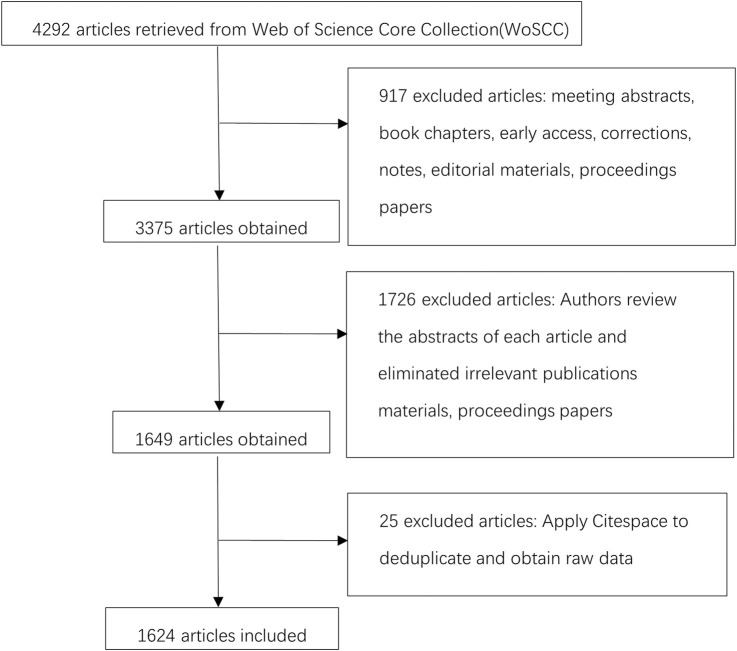
Flow chart of the study retrieval and selection process.

The WoSCC database comprised a total of 4,292 documents. After filtering for articles and reviews, 3,375 documents were obtained. Following manual thematic screening delete articles that are not related to the subject word and exporting in plain text, a total of 1,649 records meeting the criteria were gathered. After importing CiteSpace and removing duplicates, 1,624 samples were obtained. The study’s retrieval and selection process are illustrated in Figure 1. Since the data did not involve any identifiable patient information, this study did not necessitate an ethical review. Journal information, including impact factor (IF), Journal Impact Factor (JIF) quartile (Q1–Q4) and category, was collected from the 2022 Journal Citation Reports.

### 2.3 Statistical analysis

Statistical analyses were primarily conducted using CiteSpace (v6.2, R4), while VOSviewer 1.6.20 was employed for analyzing publishing journals and co-cited references statistics. CiteSpace settings included a 1-year “time slice” value, selection of different network clipping methods based on graphical complexity, the G-index factor K set to 25, and TopN% set at 10%. The configuration of image nodes and connections was tailored to the specific objective of each analysis. VOSviewer analysis was conducted using the default settings.

## 3 Results

### 3.1 Trend of global publications and citations

**FIGURE 2:**
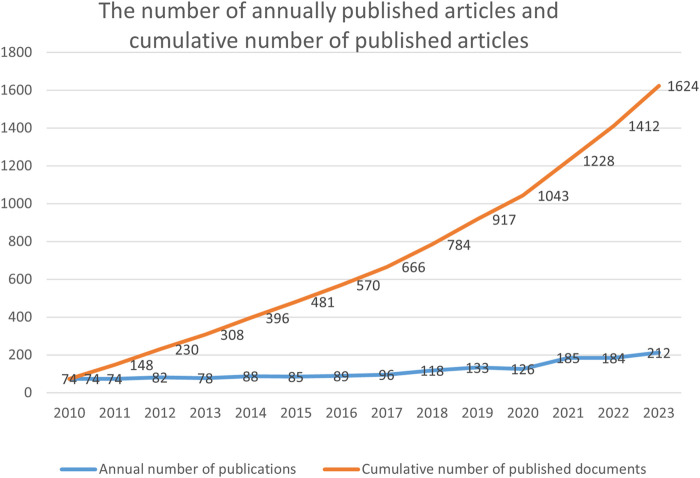
Number of annually published articles and cumulative number of published articles.

A total of 1,624 documents were included in this study. A line chart (Figure 2) was created to depict the quantity and overall count of publications on MN over the past 13 years (2010–2023), illustrating its progression. The vertical axis indicates the number of published papers, and the horizontal axis denotes the year. The total volume of publications over a certain period can objectively and quantitatively reflect the overall developmental trend of a field.

From 2010 to 2020, the growth rate of publications remained relatively stable. However, a notable increase in the number of publications occurred in 2019, reaching a peak in 2023.

### 3.2 Countries/regions analysis

**FIGURE 3:**
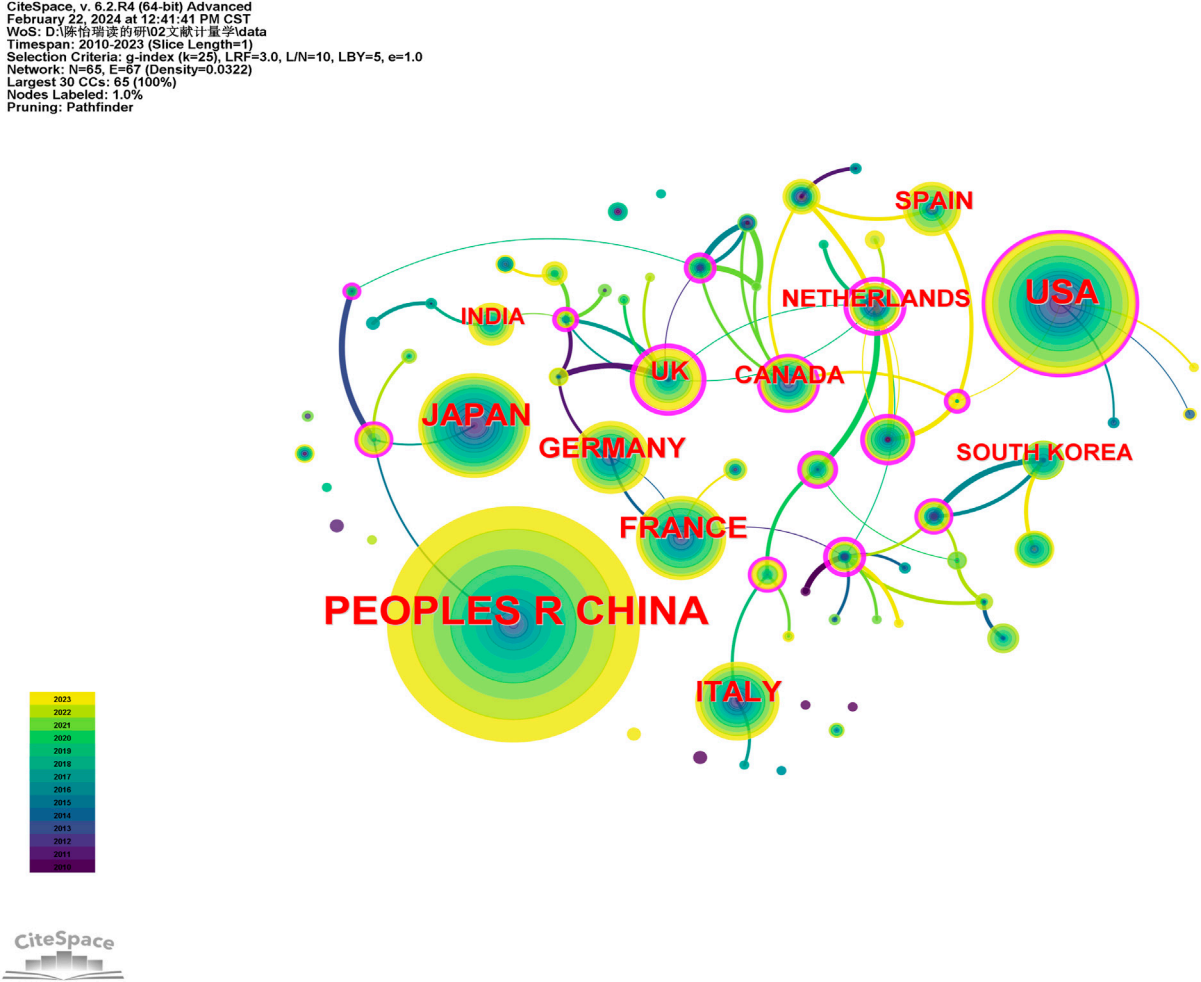
Collaboration network of countries/regions.

The articles included in this study originate from 67 different countries, with over half of the articles coming from the top three countries. China leads with the highest number of articles (708, 35.3%), followed by the United States (264, 13.2%), and Japan (151, 7.5%), as depicted in Table 1. Utilizing CiteSpace to examine country nodes, Figure 3 presents a collaborative network map among countries, revealing extensive international collaboration.

**TABLE 1:** Top 10 countries/regions in terms of publications.

Rank	Country	Publications	Centrality
1	People’s Republic of China	708	0.03
2	United States	264	0.17
3	Japan	151	0.04
4	France	110	0.06
5	Italy	92	0.17
6	Germany	84	0.11
7	England	58	0.19
8	Canada	51	0.13
9	Spain	51	0.07
10	Netherlands	42	0.11

Despite having a relatively low publication count (15, 0.007%), Sweden emerges as the country with the highest centrality (0.20). In the top 10, England (0.19), Italy (0.17), and the United States (0.17) exhibit higher centrality, underscoring their significant roles in international collaborations.

### 3.3 Institution analysis

A total of 393 institutions have published papers related to MN, with the top 10 publishing institutions outlined in Table 2 (Alphabetical order if publications and centrality are the same). The Sorbonne Universite (74, 0.08) tops the list with a high number of publications and a higher centrality than the Institut National de la Sante et de la Recherche Medicale (74, 0.04), followed by Assistance Publique Hopitaux Paris (APHP) (68, 0.01), with the leading three institutions all based in France. This highlights the significant interest and focus of French institutions in the field of MN.

**TABLE 2:** Top 10 institutions in terms of publications.

Rank	Institution	Publications	Centrality	Country
1	Sorbonne Universite	74	0.08	France
2	Institut National de la Sante et de la Recherche Medicale (Inserm)	74	0.04	France
3	Assistance Publique Hopitaux Paris (APHP)	68	0.01	France
4	Mayo Clinic	59	0.13	United States
5	Hopital Universitaire Tenon - APHP	57	0.06	France
6	Peking University	55	0.17	People’s Republic of China
7	Boston University	52	0.23	United States
8	University Medical Center Hamburg-Eppendorf	50	0.04	Germany
9	University of Hamburg	50	0.04	Germany
10	Universite Paris Cite	47	0.04	France

**FIGURE 4:**
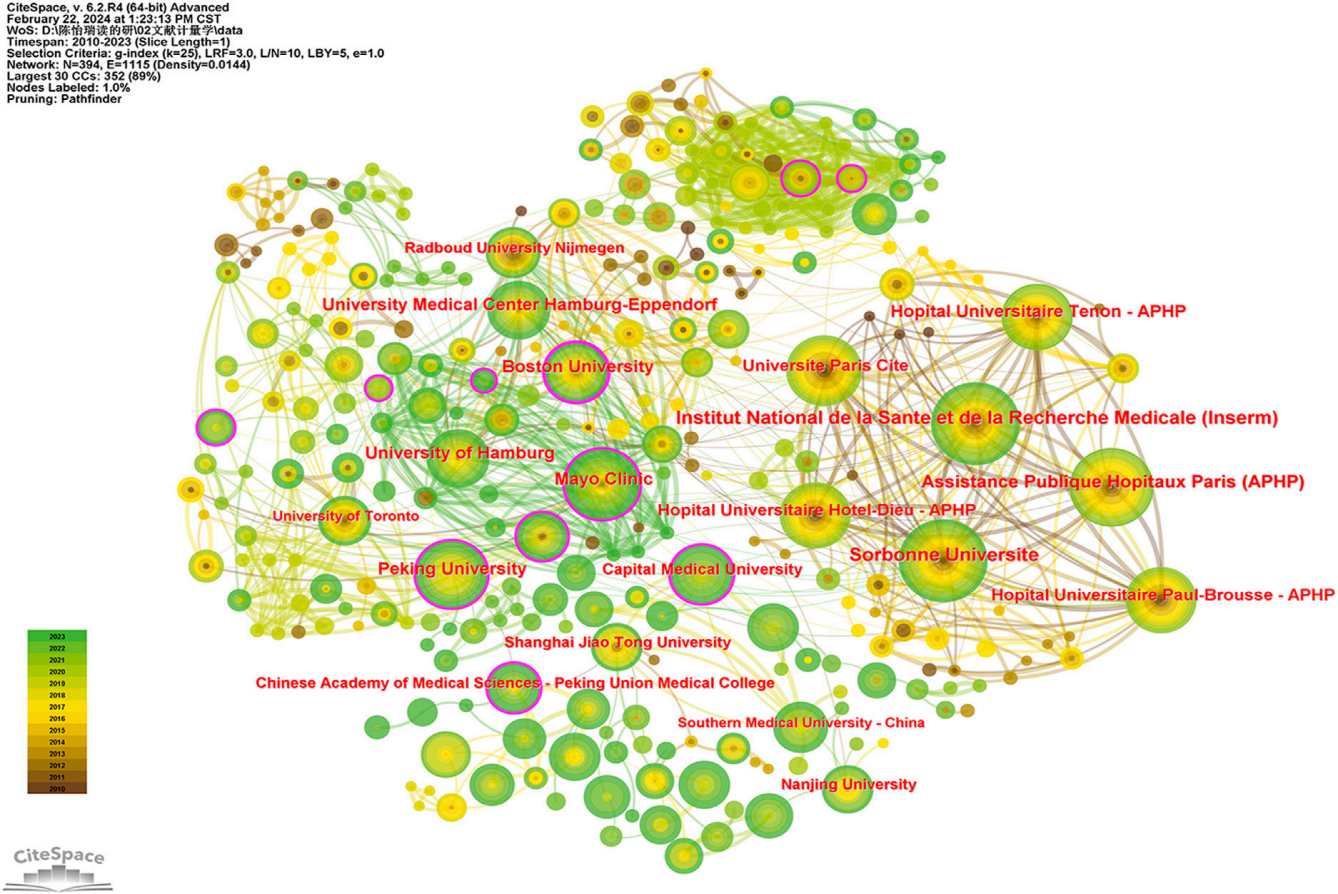
Collaboration network of institutions.

With institutions represented as nodes, an institutional co-occurrence map was created (Figure 4). Boston University (0.23) in the United States is identified as a core entity in inter-institutional cooperation, with Peking University (0.17) and Capital Medical University (0.16) also playing key roles in these collaborations. The network density of the institutional co-occurrence graph stands at 0.0144, indicating a lack of overall collaboration among institutions researching MN. Enhancing cooperation between institutions, and leveraging the unique resources and information each possesses, could significantly benefit the advancement of the field.

### 3.4 Author analysis

A total of 554 authors have made contributions to the field of MN. Table 3 (sorted alphabetically by name if the number of papers is the same) lists the 10 authors with the highest number of publications. Debiec Hanna is the most prolific author with 50 publications, followed closely by Ronco Pierre (48) and Fervenza Fernando C (35) as the second and third most prolific authors, respectively.

**TABLE 3:** Top 10 most productive authors.

Rank	Authors	Papers
1	Debiec Hanna	50
2	Ronco Pierre	48
3	Fervenza Fernando C	35
4	Beck Laurence H Jr	32
5	Wetzels Jack F M	29
6	Cui Zhao	26
7	Zhao Ming-hui	26
8	Hoxha Elion	21
9	Stahl Rolf A K	21
10	Brenchley Paul E	14

**FIGURE 5:**
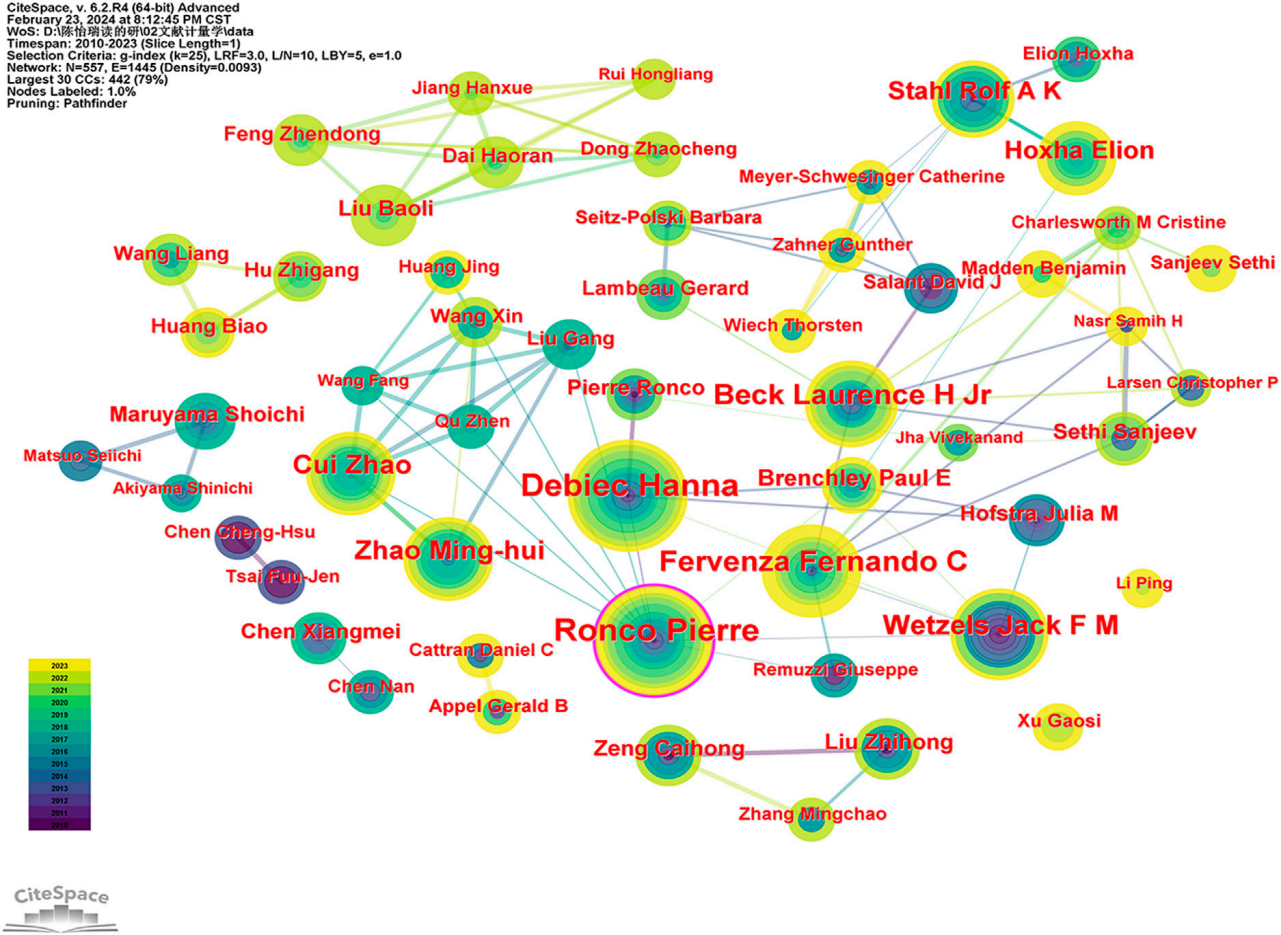
Collaboration network of authors.

Using CiteSpace, an author collaboration network graph was generated (Figure 5). According to Price’s law, the minimum number of publications required for core authors is n = 0.749 $\sqrt{Nmax}$ , where *N*max is the number of publications by the most prolific author (de Solla Price, 1963). Consequently, the minimum number of publications required for an author to be considered a core author in the field of MN research rounds to 6 articles (5.3). There are 56 core authors who have collectively published a total of 684 papers, which constitutes 37.96% of all articles. According to Lotka’s law, core authors should account for 50% of the total publications (Lotka, 1926). As such, the MN literature does not conform to Lotka’s law, indicating that the core author group in this field has not yet formed and is still evolving.

### 3.5 Journals analysis

According to VOSviewer analysis, a total of 3,714 journals have published articles relevant to the field. Table 4 displays the top 10 journals. BMC Nephrology published the most articles (78), followed by Renal Failure (63), and the Journal of the American Society of Nephrology (50). The majority of the top 10 journals fall within the nephrology category, with half of them ranked in WoS Q1. Kidney International has the highest Impact Factor (IF) at 19.6, indicating that the literature on MN is generally of high quality. Journal information, including impact factor (IF), Journal Impact Factor (JIF) quartile (Q1–Q4) and category, was collected from the 2022 Journal Citation Reports.

**TABLE 4:** Top 10 most productive journals.

Rank	Cited journals	Papers	Citations	If	JIF quartile	Category
1	Bmc Nephrology	78	684	2.2	Q3	Urology and Nephrology
2	Renal failure	63	372	3	Q2	Urology and Nephrology
3	Journal of the American Society of Nephrology	50	4,936	13.6	Q1	Urology and Nephrology
4	Medicine	48	295	1.6	Q3	Medicine, General and Internal
5	Clinical Nephrology	44	303	1.1	Q4	Urology and Nephrology
6	Nephrology Dialysis Transplantation	42	1,526	6.1	Q1	Urology and Nephrology
7	International Urology and Nephrology	42	276	2	Q3	Urology and Nephrology
8	Frontiers in Immunology	38	490	7.3	Q1	Frontiers in Immunology
9	Kidney International	37	2,351	19.6	Q1	Urology and Nephrology
10	Clinical Journal of the American Society of Nephrology	36	2,610	9.8	Q1	Urology and Nephrology

**FIGURE 6:**
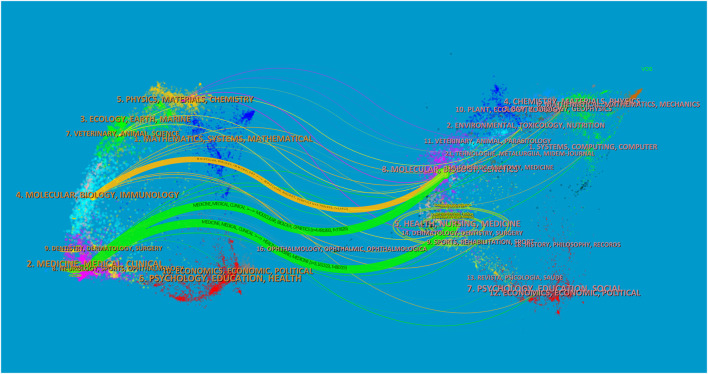
Dual-map overlay of membranous nephropathy.

Additionally, we generated a dual-map overlay with CiteSpace (Figure 6), identifying three principal citation trajectories (two in green and one in orange). These trajectories consist of a citing map on the left and a cited map on the right. The majority of studies were published in journals categorized under medicine, medical, and clinical disciplines and were commonly cited in articles related to health, nursing, and medicine.

### 3.6 Analysis of co-cited references

**FIGURE 7:**
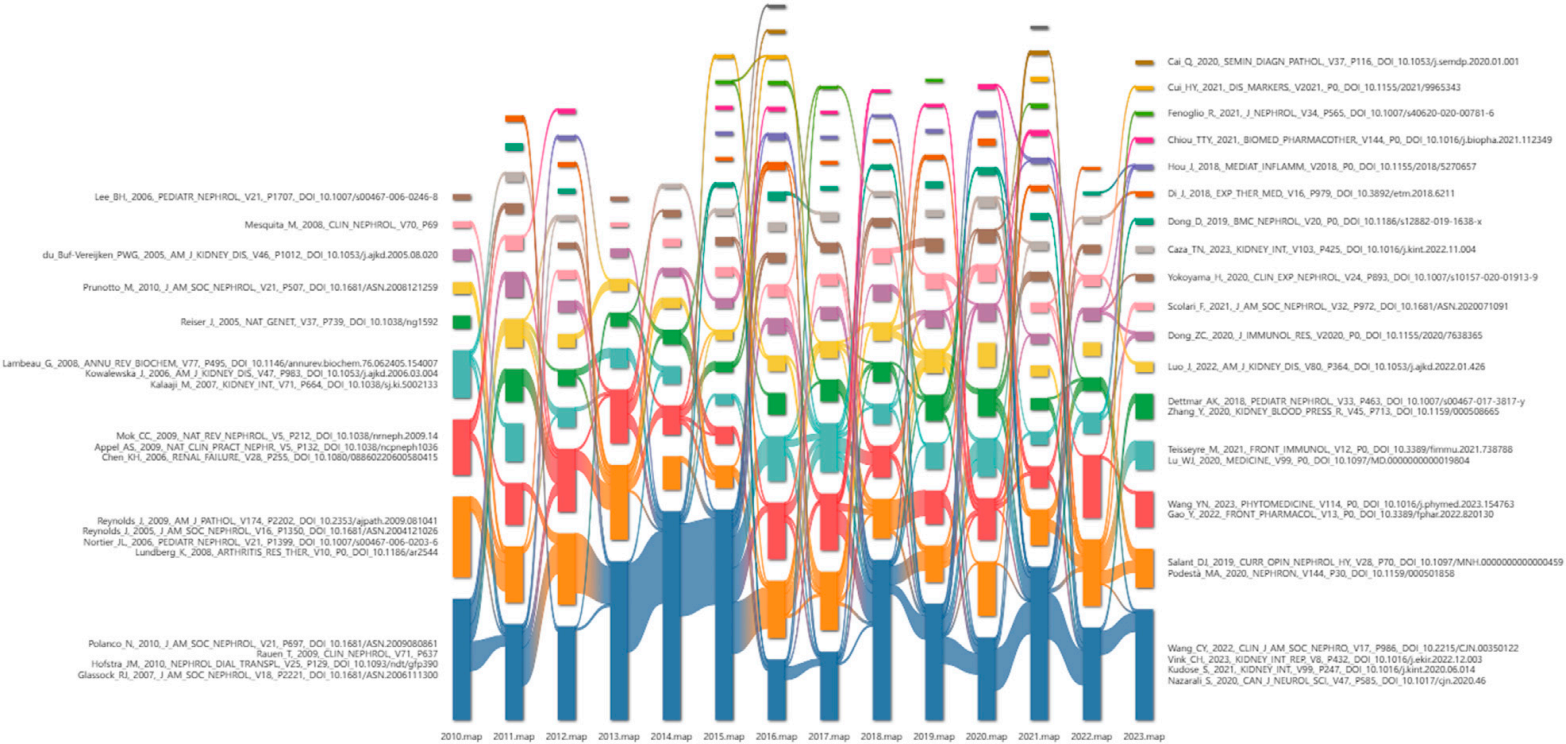
Alluvial flow map of co-cited references from 2010 to 2023.

Using CiteSpace depicts the alluvial flow map of co-cited references from 2010 to 2023 (Figure 7), revealing that certain co-cited references were extensively cited during the 2015–2023 period. Table 5 lists the ten papers with the highest co-citation intensity, encompassing five reviews and five articles. According to CiteSpace analysis, the publication with the highest co-citation intensity is “Primary Membranous Nephropathy,” a review by William G. Couser, published in the Clinical Journal of the American Society of Nephrology in 2017, receiving a total of 584 citations. This review addresses the etiology, pathogenesis, pathology, clinical manifestations, treatment, and prognosis of primary MN (Couser, 2017). The most cited article is “M-Type Phospholipase A2 Receptor as Target Antigen in Idiopathic Membranous Nephropathy” by L. H. Beck Jr, published in 2009. It identified the presence of PLA2R in the normal podocytes and immune deposits of most patients with idiopathic MN (Beck et al., 2009).

**TABLE 5:** Top 10 papers with the highest co-citation strength.

Rank	Title	Author (Year of publication)	Journal	Co-citation strength	Total citations
1	Primary membranous nephropathy	William G. Couser (2017)	Clinical Journal of the American Society of Nephrology	188	584
2	M-Type Phospholipase A2 Receptor as Target Antigen in Idiopathic Membranous Nephropathy	LH Beck Jr (2009)	New England Journal of Medicine	160	2,563
3	Thrombospondin type-1 domain-containing 7A in idiopathic membranous nephropathy	NM Tomas (2014)	New England Journal of Medicine	143	931
4	Rituximab or cyclosporine in the treatment of membranous nephropathy	FC Fervenza (2019)	New England Journal of Medicine	137	399
5	A proposal for a serology-based approach to membranous nephropathy	AS De Vriese (2017)	Journal of the American Society of Nephrology	135	344
6	Neural epidermal growth factor-like 1 protein (NELL-1) associated membranous nephropathy	S Sethi (2020)	Kidney International	123	276
7	Rituximab for severe membranous nephropathy: a 6-month trial with extended follow-up	K Dahan (2017)	Journal of the American Society of Nephrology	116	362
8	Exostosin 1/exostosin 2–associated membranous nephropathy	S Sethi (2019)	Journal of the American Society of Nephrology	107	253
9	Phospholipase A2 receptor autoantibodies and clinical outcome in patients with primary membranous nephropathy	E Hoxha (2014)	Journal of the American Society of Nephrology	106	365
10	Pathophysiological advances in membranous nephropathy: time for a shift in patient’s care	P Ronco (2015)	The Lancet	101	361

### 3.7 Analysis of keywords

**FIGURE 8:**
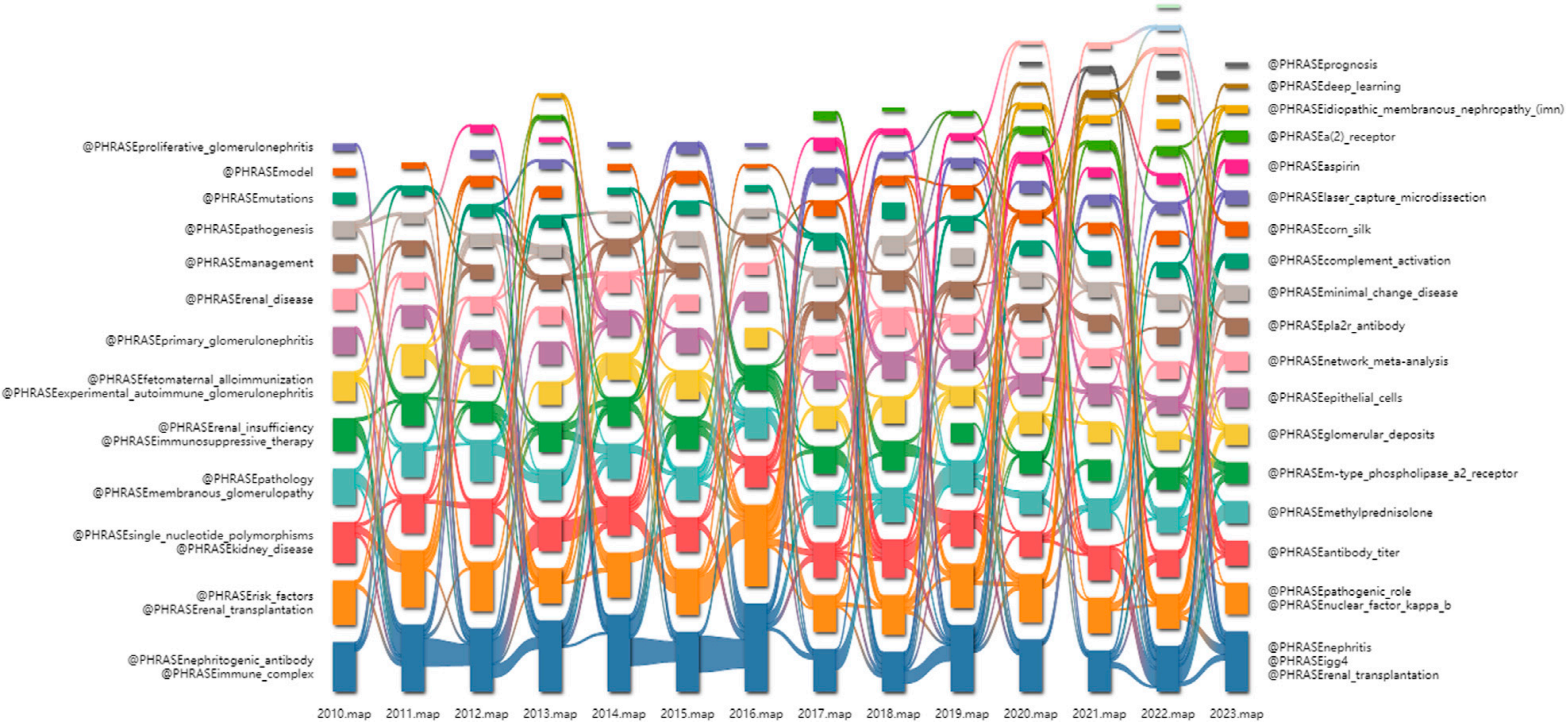
Alluvial flow map of keywords from 2010 to 2023.

Figure 8 illustrates the evolution of keywords over time via citespace. In 2010, “nephritogenic antibody” and “immune complex” were among the most frequently used keywords. As time progressed to 2023, the predominant keywords shifted to “nephritis,” “igg4,” and “renal transplantation.”

Table 6 lists the ten most frequently occurring keywords, with three of them being part of the search terms used, indicating a high degree of homogeneity in the literature related to MN. The most common keywords included “membranous nephropathy” (713), “nephrotic syndrome” (516), and “glomerulonephritis” (325).

**TABLE 6:** Top 10 keywords with the highest frequency.

Rank	Frequency	Keywords
1	713	Membranous nephropathy
2	516	Nephrotic syndrome
3	325	Glomerulonephritis
4	237	Idiopathic membranous nephropathy
5	232	Autoantibody
6	211	Receptor
7	172	Antibody
8	169	Disease
9	143	Rituximab
10	138	Phospholipase a2 receptor

**FIGURE 9:**
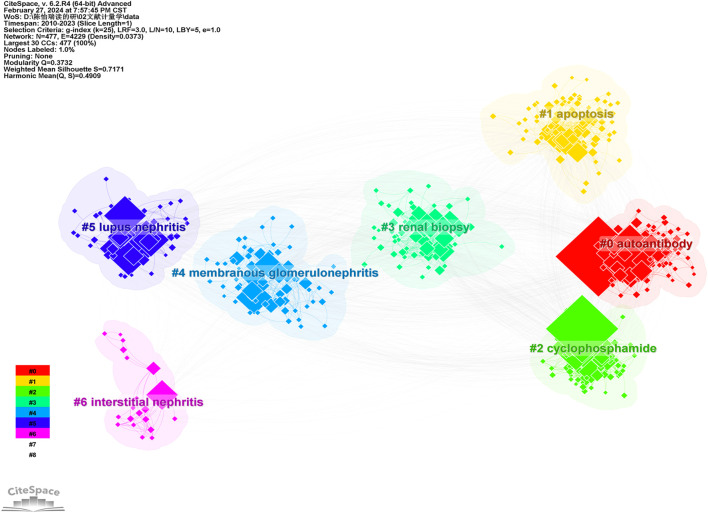
Knowledge map of keyword clustering.

The knowledge map of keyword clustering reveals seven clustering patterns within the field of MN research (Figure 9), with a modularity Q of 0.3732 and a mean silhouette value S of 0.7171. These values indicate that the keywords exhibit a diverse range of characteristics, and the clustering structure is both significant and compelling, enabling the identification of research hotspots. The primary labels for our keyword clusters include “autoantibody”, “apoptosis”, “cyclophosphamide”, “renal biopsy”, “membranous glomerulonephritis”, “lupus nephritis”, and “interstitial nephritis”.

**FIGURE 10:**
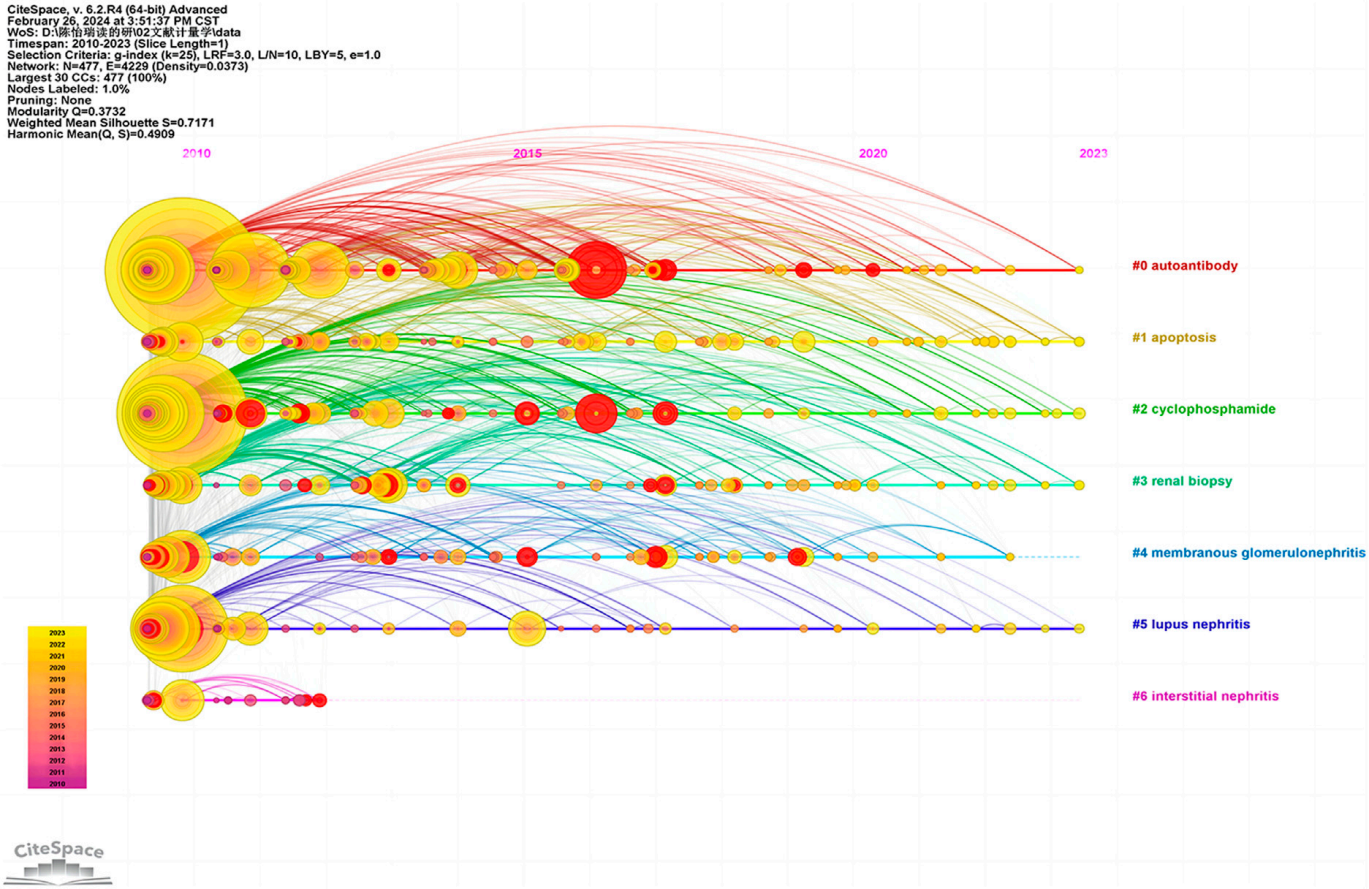
Timeline view of keyword clustering analysis.

As shown in the timeline view (Figure 10), “autoantibody”, “apoptosis”, “cyclophosphamide”, “renal biopsy” are popular keywords in recent years (2010–2023), and cluster sizes are 96, 89, 80, and 75, respectively.

**FIGURE 11:**
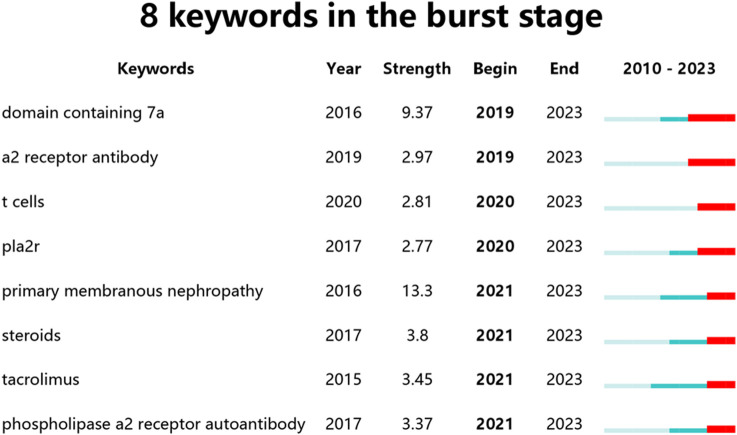
Eight keywords in the burst stage.

Recent burst keywords were selected to create eight keywords in the burst stage (Figure 11), where the red lines represent periods of keyword surges. This visualization demonstrates that “domain containing 7a,” “a2 receptor antibody,” “t cells,” “pla2r,” “primary membranous nephropathy,” “steroids,” “tacrolimus,” “phospholipase A2 receptor,” and “autoantibody” are current research hotspots and potential targets within this field.

## 4 Discussion

### 4.1 General information

This study conducted an analysis of the MN literature from 2010 to 2023. The findings indicate that the publication growth rate has remained relatively stable over the past 13 years, with a significant surge in scholarly output observed in 2019. This surge can be attributed to advancements in glomerular laser microdissection and mass spectrometry techniques, facilitating research and antigen identification (Ronco and Debiec, 2021). Based on current trends, the volume of publications is anticipated to continue its upward trajectory into 2024.

Among the top ten most productive countries in MN research, only two are from East Asia, with China being the sole representative from the developing world. Industrialized countries commonly attribute the genesis of immune disorders to environmental factors. For instance, workers in construction and those exposed to occupational toxins such as asbestos, lead, and organic solvents are considered at an increased risk for MN (Cremoni et al., 2022). While China takes the lead in publication volume, possibly due to robust economic growth and extensive industrialization, environmental protection remains a challenge that potentially explains the rising incidence of MN (Xu et al., 2016). China’s low centrality indicates potential benefits from enhanced cooperation and development in related fields, as well as the production of high-quality articles. Research on the genetic and ethnic aspects of MN is centered around Caucasian populations in Western countries, including the UK, France, and the US (Stanescu et al., 2011). This focus may explain the higher centrality values of these countries compared to China, suggesting that despite China’s significant volume of research, the international impact in terms of collaboration and citations may be more pronounced in Western nations. Genetic heterogeneity and geographic variations in allele frequencies are observed among Chinese populations, with marked differences in causal gene variants between individuals of European, East Asian, and African American ancestries (Gupta et al., 2018). The absence of third-world countries and institutions from our lists does not diminish the severity of MN in these regions. Instead, it highlights challenges such as economic constraints and limited research infrastructure. Glomerular diseases, including MN, constitute a substantial portion of chronic kidney disease in low- and middle-income countries. The epidemiological profile of MN in these countries remains largely uncharted, primarily due to limited access to nephropathology services and concerns regarding the safety of renal biopsy procedures (Ekrikpo et al., 2022).

French institutions are notably active in MN research, with the top three institutions located in France. Debiec Hanna, from Sorbonne Universite, is the most prolific author, focusing on MN research, with his latest work being “Mayo Clinic consensus report on membranous nephropathy: proposal for a novel classification” published in Kidney International in 2023. However, according to Price’s law, the core author group within the MN domain has not yet formed and is in a developmental phase.

The top ten for journals for MN publications predominantly belong to the nephrology category and are of high quality. In co-citation analysis, LH Beck Jr from Boston University authored an influential publication, cited 2,563 times, demonstrating PLA2R as the primary antigen in MN (Beck et al., 2009). Journals publishing articles on MN mainly span medicine, medical, and clinical fields, while the most cited journals predominantly cover health, nursing, and medicine.

### 4.2 Research hotspot of membranous nephropathy

A keyword analysis conducted by CiteSpace from 2010 to 2023 reveals “membranous nephropathy,” “nephrotic syndrome,” and “glomerulonephritis” as the most prominent keywords. Clusters including “autoantibody,” “apoptosis,” and “cyclophosphamide” emerge as current popular topics. Extensive clinical research indicates that approximately 70% of MN patients possess autoantibodies (Tomas et al., 2014) and the IgG4 subclass (Kim et al., 2015) against phospholipase A2 receptor 1 (PLA2R1). Additionally, antigens related to thrombospondin type-1 domain-containing 7A (THSD7A) (5%) are detected in the serum of patients with anti-PLA2R1-negative MN (Tomas et al., 2014). Breakthroughs in 2019 led to the discovery of novel antigens such as exostosins 1 and 2 (EXT1/2) (Iwakura et al., 2022; Sethi et al., 2019), neural epidermal growth factor-like 1 protein (NELL-1) (Sethi et al., 2020a; Nassar et al., 2023) semaphorin 3B (Sema3B) (Sethi et al., 2020b; Fila et al., 2022), and protocadherin 7 (PCDH7) (Sethi et al., 2021). PLA2R, THSD7A, NELL1, and SEMA3B account for 80%–90% of the target antigens in MN (Sethi, 2021).

The “apoptosis” cluster includes keywords such as podocyte injury and expression, highlighting their relevance to MN pathology. Podocytes, crucial for glomerular filtration, rely on high basal autophagy. Proteinuria in MN, primarily caused by podocyte damage, results in significant protein loss in urine. A study has demonstrated downregulation of PLA2R autophagy in MN patients, exacerbating podocyte damage, increasing urine protein levels, and subsequently impairing renal function (Yang et al., 2021). Furthermore, microRNAs such as miR-195-5p, miR-192-3p, and miR-328-5p and their target genes PPM1A, RAB1A, and BRSK1 in the peripheral blood and urine of MN patients may participate in inflammation and apoptosis (Zhou et al., 1903). Inhibition of miR-193a inhibited renal injury, podocyte injury, and tissue cell apoptosis resulting from MN (Li et al., 2019).

Keywords associated with the cluster “cyclophosphamide” mainly include cyclophosphamide, rituximab, tacrolimus, and cyclosporine, relating to treatment protocols for MN. The 2021 guidelines recommend using rituximab or cyclophosphamide and alternate-month glucocorticoids for 6 months, or tacrolimus-based therapy for ≥6 months for the “high risk” group. Cyclophosphamide and steroids are recommmended for the “very high risk” group in the 2021 guidelines (Rovin et al., 2021). In patients in remission who tested positive for anti-PLA2R antibodies, the rituximab group showed a more rapid decline in autoantibodies, both in magnitude and duration, than the cyclosporine group (Fervenza et al., 2019).

Based on the analysis of emerging keywords, “a2 receptor antibody,” “domain containing 7a,” and “t cell” are likely to remain focal points of research in the coming years. Given that MN is diagnosed pathologically, its research hotspots have consistently revolved around antibodies. It is anticipated that future studies will also predominantly focus on antibody-related research. The “kidney as a sink” hypothesis may explain the asynchronous appearance of serum PLA2R antibodies and glomerular PLA2R antigens (van de Logt et al., 2015), aligning with Xie’s findings that all serum PLA(2)R-Ab positive patients had renal PLA(2)R positivity, and patients with renal PLA(2)R negativity were also serum PLA(2)R-Ab negative (Xie et al., 2015). However, studies linking PLA2R positivity rates with environmental and genetic factors have yet to be conducted, potentially offering a new direction for treating MN at its source.

High levels of circulating PLA2Rab contribute to increased subepithelial deposition of PLA2R antibody-antigen immune complexes in podocytes, leading to more severe podocyte injury and filtration barrier disruption, which in turn results in renal function impairment and a decline in glomerular filtration rate (Levy et al., 1996; Kaga et al., 2019). Anti-PLA2R autoantibody titers can assist in diagnosing and monitoring the response to immunosuppressive treatment (Kanigicherla et al., 2013), allowing tailored clinical treatment plans and prognosis evaluation based on PLA2R titer levels (Deng et al., 2022).

PLA2R is not the only antigen implicated in MN-induced renal damage. The type 1 thrombospondin domain-containing 7A (THSD7A) antigen, identified in 2014, accounts for a minority (3%–5%) of PLA2R-negative cases (Yang et al., 2021). Up to 28% of THSD7A-positive MN patients have been diagnosed with secondary MN due to occult malignancies (An et al., 2016), with tumors discovered within 3 months post-study in 8 patients with anti-THSD7A-associated MN (Hara et al., 2019). An essential observation also indicated that the presence of THSD7A antibodies in MN patients might be associated with race, geographical location, gender, and the underlying disease (Iwakura et al., 2015).

T regulatory cells (TREG) are reduced in the serum of MN patients and significantly improved with disease-specific treatment (Ramachandran et al., 2020). Cyclophosphamide (CYC) and calcineurin inhibitors (tacrolimus and cyclosporine) can suppress T cells and their secreted cytokines; telitacicept achieves comprehensive inhibition and regulation of lymphocyte proliferation, effectively suppressing CD20-positive B cells, plasma cells, and T cells, significantly lowering the risk of forming circulating and *in situ* immune complexes. Some researchers propose that pathogenic antibodies can be eliminated through endogenous degradation systems and pathogenic B cells can be depleted through chimeric autoantibody receptor T cells (Köllner et al., 2022), which under ideal circumstances may allow targeting the mechanisms of immune diseases while preserving protective immunity.

## 5 Conclusion

This study reviewed 1,624 articles on MN published from 2010 to 2023 to ascertain the current state of research and forecast its future directions. Over these 13 years, there has been a yearly increase in publications, indicating growing interest and extensive international collaboration in this field. The People’s Republic of China emerged as the most active contributor. Among academic institutions, France’s Sorbonne Universite and Institut National de la Sante et de la Recherche Medicale (Inserm) lead in publication volume. Debiec Hanna stands out as the most prolific author. However, a stable core group of authors has not been established. BMC Nephrology boasts the highest publication count, making it the most favored journal in the discipline. The article with the greatest co-citation impact is “Primary membranous nephropathy,” a review published in 2017. Generally, the quality of articles in the MN sector is high. The most frequent keywords over the past 13 years have been “membranous nephropathy,” “nephrotic syndrome,” and “glomerulonephritis.” The analysis of emerging vocabulary suggests that “a2 receptor antibody,” “domain containing 7a,” and “t cells” may become the focal points of research in the years ahead.

### 5.1 Strengths

To our knowledge, this is the first study to offer a visual representation of MN, serving as a reference for future research directions. This research undertakes a groundbreaking visual analysis based on bibliometrics within the field of MN, aiding in the comprehension of the present research landscape and future trend directions. The combined use of CiteSpace and VOSviewer enhances the comprehensiveness and precision of the findings.

### 5.2 Limitations

Firstly, not all studies from databases were included, due to software constraints, data were exclusively sourced from the WoSCC database, and only articles published in English were considered. This introduces a potential selection bias, and the manual screening of literature could lead to information omission. Thirdly, bibliometric analysis tools come with intrinsic limitations and biases, which could impact the analysis outcomes. As mentioned above, the absence of third-world countries and institutions in the various lists does not mean that the incidence of MN is not severe in these regions. This gap may be attributable to economic factors, inadequate research facilities, etc. Further research is needed to overcome the limitations of the current study and international cooperation is critical to a global balance in MN research. Future research should lay greater stress on regions that are underrepresented, as well as those that may be less likely to publish research due to language or resource constraints.
